# The genome-wide associated candidate gene *ZNF804A* and psychosis-proneness: Evidence of sex-modulated association

**DOI:** 10.1371/journal.pone.0185072

**Published:** 2017-09-20

**Authors:** Marta de Castro-Catala, Aurea Mora-Solano, Thomas R. Kwapil, Paula Cristóbal-Narváez, Tamara Sheinbaum, Anna Racioppi, Neus Barrantes-Vidal, Araceli Rosa

**Affiliations:** 1 Secció de Zoologia i Antropologia Biològica, Departament de Biologia Evolutiva, Ecologia i Ciències Ambientals, Facultat de Biologia, Universitat de Barcelona (UB), Barcelona, Spain; 2 Institut de Biomedicina de la Universitat de Barcelona (IBUB), Barcelona, Spain; 3 Department of Psychology, University of North Carolina at Greensboro, Greensboro, North Carolina, United States of America; 4 Department of Psychology, University of Illinois at Champaign-Urbana, Champaign, Illinois, United States of America; 5 Departament de Psicologia Clínica i de la Salut, Facultat de Psicologia, Universitat Autònoma de Barcelona (UAB), Bellaterra, Barcelona, Spain; 6 Sant Pere Claver - Fundació Sanitària, Barcelona, Spain; 7 Centro de Investigaciones Biomédicas en Red de Salud Mental (CIBERSAM), Madrid, Spain; Hospital Benito Menni, SPAIN

## Abstract

**Background:**

The Zinc finger protein 804A (*ZNF804A*) is a promising candidate gene for schizophrenia and the broader psychosis phenotype that emerged from genome-wide association studies. It is related to neurodevelopment and associated to severe symptoms of schizophrenia and alterations in brain structure, as well as positive schizotypal personality traits in non-clinical samples. Moreover, a female-specific association has been observed between *ZNF804A* and schizophrenia.

**Aim:**

The present study examined the association of two *ZNF804A* polymorphisms (rs1344706 and rs7597593) with the positive dimension of schizotypy and psychotic-like experiences in a sample of 808 non-clinical subjects. Additionally, we wanted to explore whether the sexual differences reported in schizophrenia are also present in psychosis-proneness.

**Results:**

Our results showed an association between rs7597593 and both schizotypy and psychotic-like experiences. These associations were driven by females, such those carrying the C allele had higher scores in the positive dimension of both variables compared to TT allele homozygotes.

**Conclusion:**

The findings of the present study support the inclusion of *ZNF804* variability in studies of the vulnerability for the development of psychopathology in non-clinical samples and consideration of sex as a moderator of this association.

## Introduction

Genome-wide association studies (GWAS) have identified thousands of genes and genetic variants contributing to the development of complex diseases, although the biological mechanisms by which most of these genes act remains unclear. Similarly, GWAS in schizophrenia have detected numerous candidate loci (e.g.:[1,2]). One of the first genes that has achieved genome-wide level of statistical significance in schizophrenia GWAS is the Zinc finger protein 804A (*ZNF804A*). Despite the substantial genetic evidence of two single-nucleotide polymorphisms (SNPs) within this gene (i.e. rs7597593 and rs1344706, showing A and T alleles conferring increased risk for schizophrenia, respectively) [1,3], the function of the protein and the molecular mechanisms responsible for enhancing risk for psychosis were unknown.

Further research showed that this gene was related to neurodevelopment and plasticity, influencing the expression of genes involved in cell adhesion and important processes such as neural migration, neurite outgrowth and synapse formation [4,5]. ZNF804A is expressed in the brain and contains a C2H2-type domain associated with the zinc-finger protein family with a role in transcription. More recent evidences suggested that *ZNF804A* localizes to synapsis and that it plays a role in neurite formation, maintenance of dendritic spines, and activity-dependent structural plasticity [6], which are found to be altered in brains of schizophrenia patients. Additionally, the expression of *ZNF804A* has been observed to peak in both the rat and human brain during the prenatal period when neuronal migration ends and neuronal differentiation and maturation begins [7,8]. Empirical work and predictive bioinformatic analyses have suggested that the two GWAS-associated polymorphisms (rs7597593 and rs1344706) may modify the affinity of the gene sequence for DNA- and/or RNA-binding proteins, which might in turn alter the expression levels of the gene [9]. In this sense, for example, the A allele of rs1344706 has been associated with lower ZNF804A expression during the second semester of fetal brain development [8] and increased mRNA expression in post-mortem dorsolateral prefrontal cortex from psychiatrically normal controls [3]. Also rs7597593 was associated with post-mortem brain mRNA expression levels of ZNF804A, showing increased levels in C allele carrier female subjects [10].

In relation to the clinical phenotype of schizophrenia, this gene seems to influence the expression of several genes associated to the positive dimension of the illness [11,12], which includes psychotic symptoms such as hallucinations and delusions. Moreover, the A allele on rs1344706 was found related to elevated manic symptoms in psychotic patients [13], more severe symptoms in schizophrenia spectrum disorder patients [14], as well as poorer clinical outcome in first episode patients [15]. This allele has also been associated with altered brain macro- and micro-structure in healthy people, first episode patients, and patients with chronic schizophrenia or bipolar disorder [14,16–18]. The association of *ZNF804A* appears to be moderated by sex. Specifically, rs7597593 was strongly associated with schizophrenia in women, but not in men [10]. However, this sex differential associations need more attention, because the other study exploring this did not find sex moderation [3].

Convergent evidence has shown shared genetic determinants between schizophrenia and intermediate phenotypes of the disease, such as functional brain alterations, neurocognitive deficits, schizotypal personality traits or psychotic-like experiences, which are observable across the psychosis continuum (i.e. non-clinical, subclinical and clinical samples) [19,20]. In this regard, several neurocognitive and neuroimaging studies pointed out that *ZNF804A* variability was associated with altered functional connectivity, relatively less impaired neuropsychological performances and reduced activation during measures of social cognition (e.g.: [14,21].

Only two previous studies have explored the effect of the *ZNF804A* gene on psychosis proneness in non-clinical samples. The first study, observed that subjects carrying the T allele of rs1344706 were showing higher schizotypy scores than those CC, specifically in the case of the disorganization schizotypy factor [22]. A more recent study, in a large cohort of 1507 healthy young male conscripts, found that the C alleles of both rs7597593 and rs1344706 were associated to schizotypy, specifically to self-rated paranoia and ideas of reference. The rs7597593 C allele was also associated with higher perceptual aberration and positive psychotic-like experiences [23]. Both studies found an effect for this gene in their non-clinical samples, although the results reported for the rs1344706 are controversial in terms of the allele conferring risk.

Considering the contradictory findings from the above mentioned studies, the aim of the present study was to study the association between two *ZNF804A* polymorphisms (rs7597593 and rs1344706) and schizotypy and psychotic-like experiences in a sample of 808 non-clinical subjects. According to the hypothesis of the psychosis continuum, we hypothesize that *ZNF804A* variability will be associated with the positive dimension of both traits. Moreover, given the differential sex results observed in the literature with schizophrenia, we wanted to explore for the first time whether these differences between males and females were also present in psychosis proneness.

## Materials and methods

### Participants

The participants were drawn from a sample of 808 university and technical schools students from the area of Barcelona. Subjects that were of non-European origin (parents born in non-European countries) were excluded because genotyping frequencies for the studied polymorphism differ between populations. The final sample comprised 706 young adults (mean age = 20.78, SD = 4.19) from which 166 were men (23.5%) and 540 were women (76.5%). Males and females differed slightly in terms of age (males: mean = 21.4, SD = 4.7; females: mean = 20.6, SD = 4.01, p < 0.05). Ethical approval was obtained from the Universitat Autònoma de Barcelona Ethics Committee. All subjects volunteered to take part in the study and provided written informed consent at assessments, after being informed of the objectives of the study. They were not preselected based upon any criteria.

### Psychosis proneness assessment

All participants completed self-report measures assessing positive schizotypy and positive psychotic-like experiences. Schizotypy was assessed with the Spanish version of the Wisconsin Schizotypy Scales (WSS), which includes the Perceptual Aberration, Magical Ideation, Revised Social Anhedonia, and Physical Anhedonia Scales [24–28]. Exploratory and confirmatory factor analyses of the four scales reliably produce two factors, positive and negative schizotypy, that account for 80% of the variance. Magical Ideation and Perceptual Aberration Scales loaded on the positive schizotypy factor and Physical Anhedonia and Revised Social Anhedonia loaded on the negative schizotypy factor. The technical school volunteers completed the short version of the scales, which have comparable reliability and correlate highly with the original versions [29]. Also, the factor structure underlying the short scales is comparable with the factor structure of the original scales [30]. Participants were assigned positive schizotypy factor scores based upon factor loadings derived from a sample of 6137 college students [31]. The formulae for computing these scores have been used in previous studies and are described in detail in [30,32].

Positive psychotic-like experiences were assessed with the Spanish version of the Community Assessment of Psychic Experiences (CAPE) [33,34]. The CAPE is a self-report questionnaire that measures lifetime prevalence of psychotic-like experiences on a frequency scale ranging from ‘never’ to ‘nearly always’, evaluating three dimensions of symptoms: positive, negative, and depressive. It has good validity and reliability and has been used in general population studies [35]. A total sum score of the 20 items accounting for the positive dimension was used in the analyses. Note that only the positive dimension of both schizotypy and psychotic-like experiences were used to explore our hypothesis. In both scales, higher scores mean higher schizotypy or psychotic-like experiences.

### Genotyping

Genomic DNA was extracted from buccal mucosa on a cotton swab using the Real Extraction DNA kit (Durviz S.L.U., Valencia, Spain). The two intronic single nucleotide polymorphisms of the *ZNF804A* included in the study (rs7597593 and rs1344706; D’ = 0.94 r^2^ = 0.32) were genotyped using TaqMan 5’ exonuclease assay (Applied Biosystems).

The final volume was 5 μL, which contained 5 ng of genomic DNA, 2.5 μL of TaqMan Master Mix, and 0.125 μL of 40x genotyping assay (assays C_223561_10 and C_2834835_10, respectively). The cycling parameters were as follows: 95°C for 10 min followed by 40 cycles of denaturation at 92°C for 15s and annealing/extension at 60°C for 1 min. Polymerase chain reaction plates were read on an ABI PRISM 7900HT instrument and SDS v2.1 software (Applied Biosystems) was used for the genotype analysis of data. Both polymorphisms were in Hardy-Weinberg equilibrium. For accuracy of genotyping, 20% of the samples (chosen randomly) were genotyped twice, showing concordant genotypes.

### Statistical analyses

All data were processed using Stata v.13.1 (Stata/MP 13.1 for Windows, StataCorp LP, College Station, USA). Independent analyses of covariance (ANCOVA) were performed to explore the association of each SNP with positive schizotypy and positive psychotic-like experiences, adding age, sex and sample (whether they were undergraduate or technical school students) as covariates. Differences between males and females were also analysed. When ANCOVAs yielded significant effects, pairwise comparisons and analyses comparing carriers of one allele *vs* homozygotes of the other allele were computed to detail differences between genotypes. To address potential type I errors, statistical significance was determined using a permutation-based resampling procedure. Empirical adjusted p-values (p_adj_) were obtained by permuting the values of the dependent variables in the model. Permutations were performed in R 3.2.1 software [36] using lmPerm package [37].

## Results

The initial sample contained 808 subjects, from which 706 remained after excluding those of non-European origin. From this, one did not complete the WSS, one did not complete the CAPE, and genotyping failed for 49 individuals (6.9%) for rs7597593 and for 27 individuals (3.8%) for rs1344706. Descriptive statistics for the WSS (standardized factor scores) and CAPE (raw scores), as well as genotype frequencies of the two SNPs are presented in Table 1. No differences were observed in the genotypic frequencies between males and females (see Table 1).

**Table 1:** Descriptive data for the positive dimensions of schizotypy and psychotic-like experiences (mean ± standard deviation and range) and details on the genotypic frequencies for the two analysed SNPs (rs7597593 and rs1344706). Data are given for the whole sample, as well as by sex (comparisons between males and females are given in italics).

		Total	Males	Females
**Positive Schizotypy (WSS)**		-0.49 ±0.73 (-1.72–2.24)	-0.37 ±0.77 (-1.45–2.24)	-0.52 ±0.72 (-1.72–2.19)
			*t = 2*.*35 p = 0*.*01*	
**Positive psychotic-like experiences (CAPE)**		7.74 ±4.62 (0–24)	8.18 ±4.63 (0–24)	7.6 ±4.62 (0–24)
			*t = 1*.*42 p = 0*.*16*	
***ZNF804A* polymorphisms**				
**rs7597593**	*CC*	261 (39.73%)	65 (40.88%)	196 (39.36%)
	CT	304 (46.27%)	68 (42.77%)	236 (47.39%)
	TT	92 (14%)	26 (16.35%)	66 (13.25%)
			*χ*^2^ *= 1*.*5 p = 0*.*5*	
**rs1344706**	AA	262 (38.59%)	57 (35.19%)	205 (39.65%)
	CA	308 (46.36%)	80 (49.38%)	228 (44.10%)
	CC	109 (16.05%)	25 (15.43%)	84 (16.25%)
			*χ*^2^ *= 1*.*4 p = 0*.*5*	

**Fig 1:**
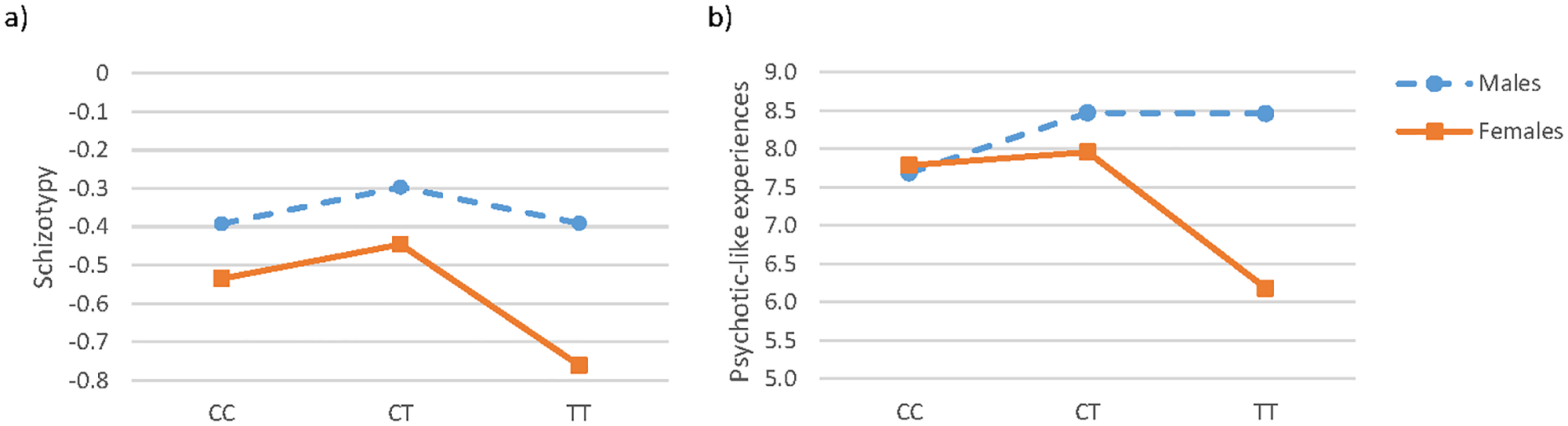
Mean scores for the positive dimension of schizotypy (a) and psychotic-like experiences (b) in relation to rs7597593 genotype in males and females.

The association analyses between rs7597593 and positive schizotypy showed a significant association in the whole sample (F = 5.06 p = 0.007 padj<0.05, Table 2). Post-hoc pairwise comparisons revealed that TT subjects were reporting lower scores than CT (t = -3.10 p = 0.006) and further analyses grouping subjects in carriers of the C allele (i.e. CC and CT genotypes) and TT homozygotes showed that those TT were reporting lower scores compared to C carriers (t = 2.45 p = 0.007). Sex-stratified analyses showed that this effect was driven by females but not males (see Table 2). In this regard, post-hoc pairwise comparisons indicated that females with the TT genotype showed lower scores than those with the CT genotype (t = -3.25 p = 0.004, Fig 1a) and further analyses revealed that females TT were reporting lower scores compared to C carriers (t = 2.87 p = 0.002).

**Table 2:** Association analyses between *ZNF804A* variation (rs7597593 and rs1344706) and the positive dimensions of schizotypy and psychotic-like experiences (mean ± standard deviation). Data is given for the whole sample, as well as by sex (F and p-values are shown in italics, * p_adj_<0.05).

		Total	Males	Females
**rs7597593**				
**Schizotypy**	CC	-0.50 ±0.75	-0.39 ±0.84	-0.54 ±0.72
	CT	-0.41 ±0.75	-0.30 ±0.74	-0.45 ±0.75
	TT	-0.66 ±0.66	-0.39 ±0.73	-0.76 ±0.61
		*F = 5*.*06 p = 0*.007*	*F = 0*.*22 p = 0*.*81*	*F = 5*.*37 p = 0*.005*
**Psychotic-like experiences**	CC	7.76 ±4.67	7.69 ±3.86	7.79 ±4.91
	CT	8.07 ±4.94	8.47 ±5.48	7.96 ±4.78
	TT	6.83 ±3.77	8.46 ±4.16	6.18 ±3.42
		*F = 4*.*07 p = 0*.02*	*F = 1*.*01 p = 0*.*67*	*F = 5*.*05 p = 0*.007*
**rs1344706**				
**Schizotypy**	AA	-0.46 ±0.74	-0.32 ±0.76	-0.50 ±0.73
	CA	-0.52 ±0.72	-0.41 ±0.74	-0.55 ±0.72
	CC	-0.44 ±0.77	-0.27 ±0.90	-0.49 ±0.72
		*F = 0*.*48 p = 0*.*62*	*F = 0*.*58 p = 0*.*56*	*F = 0*.*16 p = 0*.*85*
**Psychotic-like experiences**	AA	7.84 ±4.85	8.37 ±4.88	7.69 ±4.84
	CA	7.64 ±4.44	8.25 ±4.84	7.43 ±4.28
	CC	7.87 ±4.91	7.83 ±3.48	7.88 ±5.27
		*F = 0*.*01 p = 0*.*99*	*F = 0*.*52 p = 0*.*60*	*F = 0*.*15 p = 0*.*86*

The analyses between rs7597593 and positive psychotic-like experiences showed a significant association in the whole sample (F = 4.07 p = 0.02, Table 2). Post-hoc pairwise comparisons showed that TT subjects were scoring lower than CT (t = -2.82 p = 0.02) and further analyses grouping subjects in carriers of the C allele (i.e. CC and CT genotypes) and TT homozygotes showed that those TT were reporting lower scores compared to C carriers (t = 2.09 p = 0.02). Sex-stratified analyses between this SNP and psychotic-like experiences revealed that rs7597593 was associated in females, but not in males (Table 2). In this regard, post-hoc pairwise comparisons indicated that females with the TT genotype were showing lower positive psychotic-like experiences scores than CT (t = -3.14 p = 0.005) and CC (t = -2.72 p = 0.02, Fig 1b). Further analyses showed that females with the TT genotype were reporting lower scores compared to those carrying the C allele (t = 2.75 p = 0.003).

Regarding rs1344706, no association was observed with schizotypy or psychotic-like experiences, neither in the whole sample nor by sex (Table 2).

## Discussion

In the present study, we aimed to provide further evidence for the implication of *ZNF804A* gene variation (i.e. rs7597593 and rs1344706) on psychosis proneness and explore, for the first time, whether sex was playing a role on this association as suggested by a previous study in schizophrenia. Our main result is that non-clinical females T homozygotes for rs7597593 were reporting lower positive schizotypy and psychotic-like experiences scores compared to C carriers, whereas this was not detected in males.

Given the strong association detected between genetic variants in the *ZNF804A* in schizophrenia GWAS [1,3,38], considerable efforts have been focused on exploring the genetic variation within this gene and its influence on schizophrenia and the broader psychosis phenotype, as well as its biological mechanisms and neuronal functions (see [9,39]). For example, the GWAS associated alleles (i.e. rs1344706 A allele and rs7597593 T allele), apart from being replicated in independent case-control studies (e.g. [40]), have been found related to higher and severe clinical symptoms [13,14], worst outcome in first episode patients [15] and brain alterations (e.g. [14,16]).

Convergent evidence from family studies have shown a phenotypic relationship between levels of schizotypy or psychotic-like experiences and schizophrenia that can be attributed to shared genetic effects, which points towards a significant overlap between the underlying genetic factors inducing both psychosis proneness and schizophrenia [41]. In this sense, despite all the new studies with *ZNF804A*, the variability within this gene has been understudied in relation to psychosis proneness. Only two previous studies have explored the implication of *ZNF804A* on psychosis-related (or attenuated) traits in non-clinical samples. In the first study, Yasuda and colleagues showed that subjects carrying the T allele of rs1344706 were reporting higher schizotypy scores (particularly for the disorganized factor) in a healthy Japanese sample [22]. In contrast, in the other study exploring this, Stefanis and colleagues observed higher schizotypy scores (specifically paranoia and ideas of reference) in rs1344706 C carriers. One possible explanation for this discordant result in terms of risk allele, could be the different population origin of both samples (i.e. Japanese and European). To date, converging data suggest that *ZNF804A* is undoubtedly a risk gene for the psychotic phenotype in populations of European ancestry, but this is not clear for Asian populations. An study comparing LD patterns of the genomic region covering *ZNF804A* between Asians and Europeans showed sharp differences supporting the genetic heterogeneity, probably because differential population histories [42].

As regards to the other polymorphism studied here (rs7597593), our results are in line with those of Stefanis and colleagues reporting that subjects with the C allele showed higher scores in schizotypy, perceptual aberration and positive psychotic-like experiences.

Regarding the differences between males and females in relation to *ZNF804A* genetic variability, to the best of our knowledge, this is the first study exploring this in psychosis proneness. In our sample, we detected a female-specific association between rs7597593 and both schizotypy and psychotic-like experiences. This result expand upon Stefanis and colleagues by including females, although, contrary to them, no significant results were found in the males of our sample.

In schizophrenia, the published studies exploring this sex-specific associations have shown discordant results. Zhang et al detected a strong association between the rs7597593 T allele and schizophrenia in females of European ancestry in their sex-stratified analyses, and a trend towards interaction between sex and this SNP in their schizophrenia case-control [10]. However, no significant interaction was detected in an Irish study by Riley et al (i.e. rs17508595, rs13393273, rs7597593 and rs1344706) [3] or in a recent Chinese case-control (i.e. rs1344706) [43]. In this regard, as Zhang and colleagues pointed out that both the Irish and Chinese samples were predominately male, which might have influenced the results given the analyses of female subjects could have had less statistical power to detect the association. In our study, the predominantly female sample has allowed us to detect the *ZNF804A* by-sex effect in psychosis proneness, although the lower number of males included is a limitation that might have influenced our results.

The results of the present study support the idea of a shared underlying aetiology for schizophrenia and related attenuated phenotypes present at the different levels of severity [44,45], although different alleles have been associated in clinical and non-clinical studies. As mentioned in Stefanis et al [23], this discordance may be an example of an allelic alteration that increase risk in one population but is protective in another, as has been observed in autism, in which genetic variation affecting the balance of glutamate receptor protein synthesis and producing either an increased or diminished synthesis results in a similar deleterious effect responsible of the phenotype. Additionally, the effect of a specific variant, such as the one studied here, may depend on the constellation of many other genetic variants with small effects, as well as other factors as environmental of personal factors. In this regard, however, it is difficult to determine the biological mechanisms underlying the associations observed, because the function of the *ZNF804A* gene is still not well known. Zinc finger domains are relatively small protein motifs containing multiple finger-like protrusions that make tandem contacts with their target molecule (e.g. DNA, RNA, protein, lipid substrates), having a function in gene transcription, translation, mRNA trafficking, cytoskeletal organization, protein folding and chromatin remodelling, among others. Their binding properties depend on the amino acid sequence of the finger domains, on the linker between fingers, the number of fingers, as well as on higher-order structures. Although the role of the ZNF804 protein is not clear, variation within the *ZNF804A* may affect its binding to the target and compromise the pathways to which they are involved, including its own mRNA expression [46,47], as seem to suggest recent neuroimaging studies [48,49]. The *ZNF804A* influences the expression of three genes involved directly in dopaminergic transmission (i.e. *DRD2* and *COMT*) and cAMP signalling (i.e. *PDE4B*), two pathways thought to underlie many of the symptoms of psychosis [11]. Determining the genes regulated by *ZNF804A* may help to understand the function of this gene and how it may be involved in psychopathology. In this sense, *ZNF804A* was associated with positive schizotypy and psychotic-like experiences in the present study. Positive symptomatology has been related to hyperactivity of subcortical dopamine transmission, in which *DRD2* and/or *COMT* could be involved [50,51]. Interestingly, for both *DRD2* and *COMT*, gender-specific effects on psychosis have also been reported (e.g. [52,53]), as Zhang [10] and our study in psychosis proneness have found with *ZNF804A*. Also, as reviewed by Godar and Bortolato [54], sexual hormones (i.e. estrogens and androgens) influence differentially the development of schizophrenia-related symptoms hypothetically through the mediation of dopaminergic neurotransmission. It is tempting to speculate, thus, that *ZNF804A* could have an effect on positive symptoms through a dopaminergic-related pathway that may be influenced by sexual hormones [55]. Further functional experiments together with bioinformatics analysis might help with our understanding of these results.

The findings of the present study support the involvement of *ZNF804* variability on the vulnerability for the development of psychopathology at a non-clinical level and the implication of sex in this association. However, elucidating the mechanisms by which this gene affects mental health will be relevant in the near future.
